# Consumption of the cell-free or heat-treated fractions of a pitched kefir confers some but not all positive impacts of the corresponding whole kefir

**DOI:** 10.3389/fmicb.2022.1056526

**Published:** 2022-11-24

**Authors:** Benjamin C. T. Bourrie, Andrew J. Forgie, Tingting Ju, Caroline Richard, Paul D. Cotter, Benjamin P. Willing

**Affiliations:** ^1^Agricultural Food and Nutritional Science, Agriculture/Forestry Center, University of Alberta, Edmonton, AB, Canada; ^2^Teagasc Food Research Centre, Fermoy, Ireland; ^3^APC Microbiome Ireland, Cork, Ireland; ^4^VistaMilk, Cork, Ireland

**Keywords:** kefir, cholesterol, metabolic health, cardiovascular disease, non-alcoholic fatty liver disease

## Abstract

**Introduction:**

Kefir consumption can have many metabolic health benefits, including, in the case of specific kefirs, improvements in plasma and liver lipid profiles. Our group has previously shown that these health benefits are dependent on the microbial composition of the kefir fermentation, and that a pitched kefir (PK1) containing specific traditional microbes can recapitulate the health benefits of a traditional kefir. In this study we investigated how different preparations of kefir impact cholesterol and lipid metabolism and circulating markers of cardiovascular disease risk and determine if freeze-drying impacts health benefits relative to past studies.

**Materials and methods:**

Eight-week-old male and female C57Bl/6 mice were fed a high fat diet (40% kcal from fat) supplemented with one of 3 freeze-dried kefir preparations (whole kefir, cell-free kefir, or heat-treated kefir) for 8 weeks prior to analysis of plasma and liver lipid profiles, circulating cardiovascular disease (CVD) biomarkers, cecal microbiome composition, and cecal short-chain fatty acid levels. These groups of mice were compared to others that were fed a control low-fat diet, control high fat diet or high fat diet supplemented with milk, respectively.

**Results:**

All kefir preparations lowered plasma cholesterol in both male and female mice, while only whole kefir lowered liver cholesterol and triglycerides. Plasma vascular cell adhesion molecule 1 (VCAM-1) was lowered by both whole kefir and heat-treated kefir in male mice but not females, while c-reactive protein (CRP) was unchanged across all high fat diet fed groups in males and females.

**Conclusion:**

These results indicate that some of the metabolic benefits of consumption of this kefir do not require whole kefir while also indicating that there are multiple compounds or components responsible for the different benefits observed.

## Introduction

Humans have been utilizing fermentation as a means of food preservation for thousands of years (Hutkins, 2018). One of the most common substrates for fermentation is milk, with foods such as yogurt and kefir among the oldest fermented foods known (Bourrie et al., 2016; Hutkins, 2018). Kefir in particular is gaining popularity, largely due to an increased appreciation of the potential health benefits. Benefits that have been attributed to specific kefirs include anti-cancer (de Moreno de LeBlanc et al., 2006; Maalouf et al., 2011), immunomodulatory (Vinderola et al., 2005; Lee et al., 2007), cholesterol lowering (Bourrie et al., 2018, 2021), and other metabolic health improvements (Al-Shemmari et al., 2018; Ghizi et al., 2021).

Recently, interest in the health benefits of fermented foods has extended beyond the whole foods to consider the contribution of different fractions and even individual components of the food matrix. Indeed, many food fermentations result in the transformation of food constituents and the production of bioactive components (Marco et al., 2017). Kefir is one of the fermented foods that has had health benefits attributed to multiple different components, which not only includes the fermentation by-products but also a consortium of live microorganisms (yeast and bacteria) that could directly affect the gastrointestinal landscape. For instance, the exopolysaccharides produced by kefir specific microorganisms have been shown to have anti-obesity and immunomodulatory activity (Vinderola et al., 2006a; Lim et al., 2017), while cell-free fractions of kefir have also been shown to have immunomodulatory, lipid lowering, and anti-cancer effects (Vinderola et al., 2006b; Gao et al., 2013; Santanna et al., 2017). Additionally, kefir peptides and peptide fractions have been shown to have a wide range of health impacts including reductions in hyperlipidemia, atherosclerosis, improved neurological function, and reduced non-alcoholic fatty liver disease (NAFLD) risk (Quiros et al., 2005; Chen et al., 2016; Tung et al., 2017, 2020; Malta et al., 2022). There is also some evidence that kefir or kefir components may improve factors associated with atherosclerosis and cardiovascular disease (CVD) development, both of which are associated with obesity and hyperlipidemia (Santanna et al., 2017; Tung et al., 2020; Ghizi et al., 2021).

While traditional kefir is made using kefir grains, pitched kefir is produced by pitching pure cultures microorganisms into milk prior to fermentation. Our group has previously shown that a pitched kefir produced using microorganisms isolated from a traditional kefir could recapitulate the health benefits of traditional beverage in a mouse model of diet-induced obesity (Bourrie et al., 2021). Given that our previous work only examined the impact of whole kefir, we set out to determine whether the microorganisms present in our pitched kefir (PK1), or their components are required for lipid lowering, and if this lipid lowering effect was accompanied by concomitant improvements in CVD and atherosclerosis biomarkers such as vascular cell adhesion molecule 1 (VCAM-1) and C-reactive protein (CRP). To examine this, we fed a high-fat/high-cholesterol diet supplemented with PK1, a centrifuged and filter sterilized cell-free fraction (CFK), and a heat-treated fraction (HK) that underwent a high heat treatment to kill the kefir microbes. We also wanted to determine if freeze-dried kefir consumption would result is health benefits similar to those observed using liquid kefir in our past work. The CFK preparation allowed us to remove all microbes from the kefir and test a fraction that contained no microbes or microbial components, while the HK preparation allowed us to test a fraction in which the microbes had been killed but their microbial components were still present. By comparing how these different kefir preparations impacted weight gain, plasma and liver lipid profiles, short chain fatty acid production, gastrointestinal microbiome composition, and circulating biomarkers for CVD, we hoped to gain insight into how microbial fermentation byproducts may impact the previously described health benefits of our pitched kefir product. Sex differences that may alter the health impacts of PK1were determined by including both male and female mice. We hypothesized that the whole, heat-treated, and filter-sterilized kefir fractions share a common fermentation metabolite that improves plasma and liver lipid profiles, as well as VCAM-1 and CRP.

## Materials and methods

### Kefir grain sourcing, microbial composition, and production

Kefir fermentation was carried out as previously described for PK1 pitched kefir (Bourrie et al., 2021). Briefly, bacterial and yeast cultures were pitched into 2% fat milk and fermented at room temperature (22°C) for 18 h. Cell-free kefir was produced through centrifugation (3,000 x g for 20 min at 4°C) followed by filter sterilization using a 0.22 μm filter, while heat-treated kefir was produced by heating kefir at 85°C for 40 min. Both cell-free and heat-treated kefir were produced from freshly fermented PK1 kefir.

### Animals and treatments

A 48-week-old C57BL/6 female and 48-week-old C57BL/6 male mice were obtained from Jackson Labs. Mice were separated by sex and randomly grouped into 20 cages with 4 mice per cage by a blinded lab animal technician and groups were balanced for average body weight. All mice were housed on aspen wood chip bedding in filter-topped cages with nestlets, tunnels, and nesting material as enhancements. Room conditions, and access to food and water were carried out as previously described (5). Cages were allocated to one of 5 groups (*n* = 8) consisting of low-fat diet + milk (LFD), high fat diet (HFD) + milk (HFD), HFD + PK1 (PK1), HFD + cell-free PK1 (CFK), and HFD + heat-treated PK1 (HK). The LFD group received standard rodent chow (PicoLab Rodent Diet 5L0D), while for HFD treatment, mice received a diet consisting of 40% calories from fat supplemented with 1.25% cholesterol by weight (Research Diets D12108C). All food was provided in powdered form and supplied in food cups. Freeze-dried kefir was mixed into the food daily at a ratio of 200 mg freeze-dried kefir, equal to 2 g of liquid kefir, to 20 g of food. This would result in mice consuming kefir at a rate which equates to approximately 1 cup of kefir for a human on a 2000 kcal per day diet (a physiologically relevant dose for human consumption). This would represent approximately 0.4 kcal, or 2.5% of the daily calorie intake of the mice. Body weights were taken weekly for the duration of the study. After 8 weeks, the animals were euthanized by CO_2_ asphyxiation and blood, tissues, and cecal content were collected, snap-frozen, and stored at -80^о^C until further analysis. All experiments were carried out following the Canadian Council on Animal Care guidelines with approval from the Animal Care and Use Committee at the University of Alberta (AUP 00000671).

### Freeze-drying of kefir and microbial viability

Kefir preparations and milk underwent freeze-drying in a VirTis Ultra 35 l Freeze Dryer at an average pressure of 13mTorr and condenser temperature of −86°C for 48 h. Freeze-dried kefir and milk powder was stored at 4°C until use. Freeze-dried kefir powder was reconstituted in water and plated weekly to assess microbial survival and consistency across the trial. Bacterial enumeration was carried out using De Man, Rogosa and Sharpe (MRS) agar supplemented with 200 ppm cycloheximide while yeasts were quantified using yeast extract, glucose, and chloramphenicol (YEGC) agar. Both bacterial and yeast enumeration were carried out weekly in triplicate. Reconstituted freeze-dried milk, cell-free kefir, and heat-treated kefir had no colony growth present when plated, while the microbial density of reconstituted freeze-dried PK1 was 2.6 ± 0.4 × 10^7^ CFU/ml for bacteria and 7.0 ± 2.4 × 10^5^ CFU/ml for yeast and fresh PK1 control had 3.1 ± 0.8 × 10^8^ CFU/ml bacteria and 5.4 ± 1.9 × 10^6^ yeast. No significant differences were observed in CFU/mL of either bacteria or yeast in the freeze-dried PK1 for the duration of the study.

### Plasma cholesterol and CVD biomarker measurements

Plasma was separated from whole blood and total cholesterol was determined as previously described (Bourrie et al., 2018). High density lipoprotein-cholesterol (HDL-C) was determined using an enzyme-linked immunosorbent assay (ELISA) following manufacturer’s recommendations (MyBioSource, San Diego, CA). Non-HDL cholesterol was determined by subtracting HDL-C from total cholesterol. Plasma CRP and VCAM-1 were analyzed using commercial kits following manufacturer’s recommendations (Meso Scale Discovery, Rockville, MD; and Millipore Sigma, Burlington, MA respectively). Plasma was diluted 2,500 fold for CRP quantification and 100,000 fold for VCAM-1 quantification. Samples were measured in duplicate with CV values under 3% for both CRP and VCAM-1.

### Liver triglyceride analysis

Liver lipids were extracted using a chloroform methanol extraction, and triglycerides and cholesterol were quantified using commercial kits as previously described (Bourrie et al., 2018).

### Short-chain fatty acid analysis

Cecal content samples were thawed on ice, weighed (30 mg/sample) and homogenized in 600 μl of 25% phosphoric acid. Samples were centrifuged at 15,000 rpm at 4°C for 10 min and the supernatant was syringe filtered using a 0.45 μm filter (Fisher Scientific, Ottawa, ON). A 200 μl aliquot of filtered sample was combined with 50 μl of internal standard (23 mmol/ml, isocaproic acid) and analyzed on a Scion 456-GC instrument (Forgie et al., 2019).

### Cecal microbiota analysis

Total DNA was extracted from cecal content samples using the QIAamp fast DNA mini stool kit (Qiagen, Valencia, CA). Bacterial 16S rRNA gene amplicons were constructed using the V3V4 primer region as described by Illumina (Illumina Inc., San Diego, CA): 341F (5′- TCGTCGGCAGCGTCAGATGTGTATAAGAGACAGCCTACGGGNGGCWGCAG - 3′) and 805R (5’-GTCTCGTGGGCTCGGAGATGTGTATAAGAGACAGGACTACHVGGGTATCTAATCC-3′). Sequencing was accomplished using a paired-end MiSeq platform (2 × 300 cycles; Illumina Inc.) and raw sequences were filtered, trimmed (forward – 270 bp; reverse – 220 bp) and merged with the divisive amplicon denoising algorithm (DADA2) into amplicon sequence variants (ASVs) using the Quantitative Insight into Microbial Ecology 2 (QIIME2) pipeline (Bokulich et al., 2018; Bolyen et al., 2019); ~10,000 merged ASVs were generated per sample. Phylogenetic trees were constructed using qiime alignment (mafft; mask) and phylogeny (fastree; midpoint-root) functions. Taxonomy was assigned using the SILVA v138 database (Quast et al., 2013). QIIME2 files were transferred into R using the qiime2R (version 0.99.4) package and analyzed with the phyloseq (version 1.34.0) package (McMurdie and Holmes, 2013). Additional filtering was done to remove “Chloroplast” and “Mitochondria” assigned ASVs. The ASVs were numbered from most to least abundant. Samples were rarefied for alpha and beta diversity analyzes for females at 13,706 and males at 10,255 reads. Statistical significance was evaluated using an ANOVA with Tukey correction for Chao1, Shannon, and PD whole tree indices. Principal coordinates analysis of weighted and unweighted UniFrac indices were plotted and analyzed using the ‘betadisper’ and pairwise Adonis2 functions.

### Statistical analyzes

All data are presented as mean values with their standard errors. The level of significance for all analyzes was set at *p* < 0.05, and *p* values between 0.05 and 0.1 were considered as trends. Sample size was determined by power analysis with a statistical power of 80% and a two-sided significance level of 0.05 using an effect size of a 20% reduction in plasma total cholesterol. This analysis resulted in a total population of *N* = 40 mice with a sample size of *n* = 8 mice per group for each sex. Data were tested for normality using the Shapiro–Wilk test, while homogeneity of variance was tested using the Bartlett test. Data were tested for cage effect using the PRCOMP function in SAS and no significant cage effects were observed. Plasma cholesterol, VCAM-1, CRP, and liver lipid data was analyzed using Analysis of Variance (ANOVA) with Tukey post-hoc for multiple comparisons utilizing the R packages multcompView, ggplot2, plyr, and lmPerm. Effect of treatment on microbiota was determined using permutational multivariate analysis of variance (ADONIS).

## Results

### Whole, cell-free, and heat-treated kefir all reduced weight gain in male but not female mice

**Figure 1:**
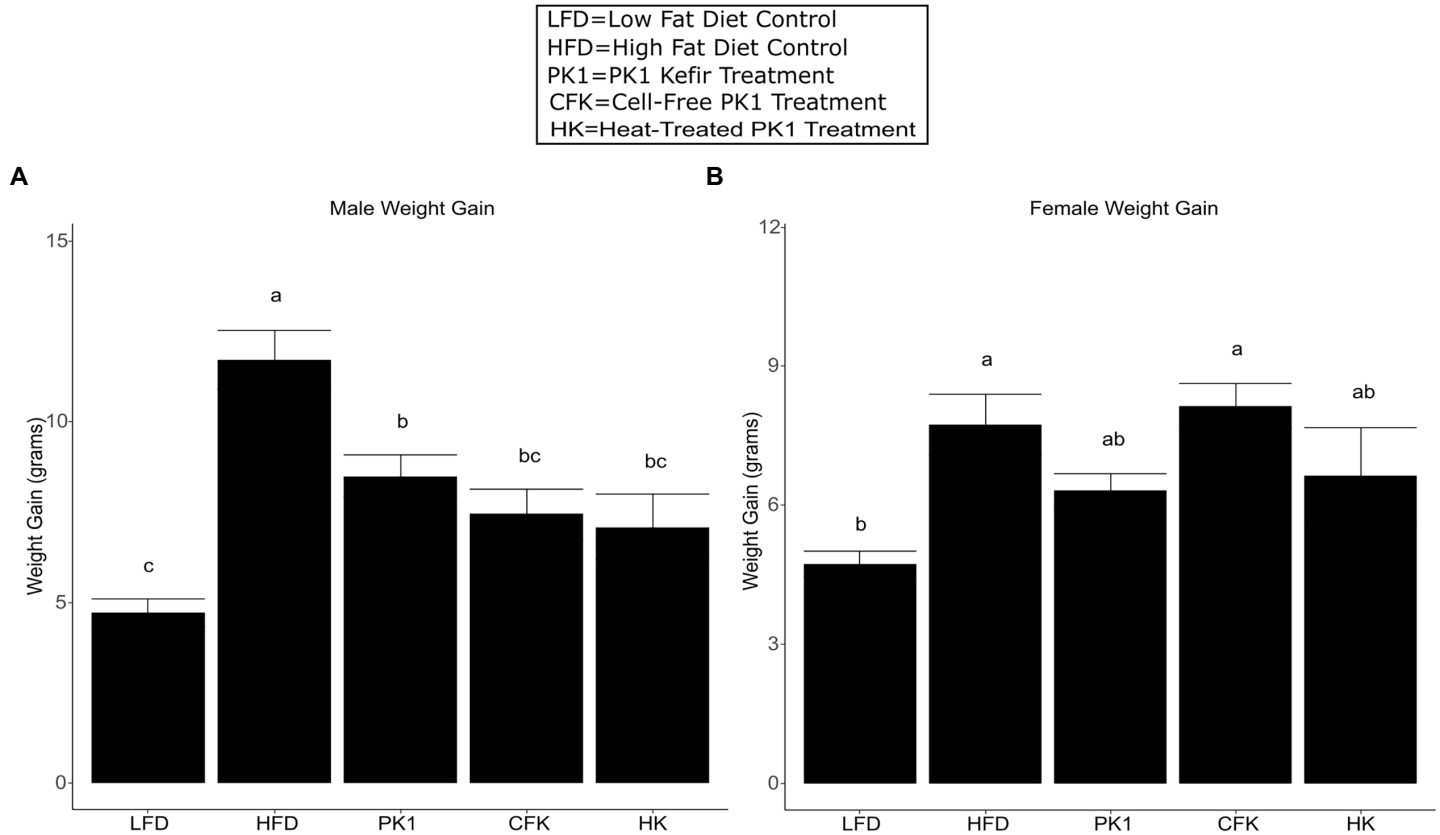
Weight gain of male **(A)** and female **(B)** C56Bl/6 mice fed a standard chow control (LFD), and (HFD) and kefir fractions on a HFD background (CFK, HK, and PK1). Data are expressed as mean values with their standard errors (*n* = 7–8). Means that do not share a letter are significantly different (*p* < 0.05). LFD, mice fed a low fat diet supplemented with freeze-dried milk for 8 weeks; HFD, mice fed a high fat diet supplemented with freeze-dried milk for 8 weeks; PK1, mice fed a high fat diet supplemented with freeze-dried pitched kefir for 8 weeks; CFK, mice fed a high-fat diet supplemented with freeze-dried cell-free kefir; HK, mice fed a high-fat diet supplemented freeze-dried heat-treated kefir.

As previous studies from our group showed inconsistent results regarding weight gain in female HFD fed mice, we wanted to examine if there were any sex specific differences in how kefir consumption and the microbial fraction of kefir impacted weight gain. After 8 weeks of high fat diet feeding, each of the PK1, CFK, and HK groups showed a reduction in weight gain in male C57Bl/6 mice (*p* < 0.05; Figure 1A) when compared to the HFD control group. In female mice, HFD and CFK mice gained more weight than LFD control, while kefir and HK had intermediate weight gain and were not different from LFD or HFD (Figure 1B).

### All kefir fractions improved plasma cholesterol profiles

**Figure 2:**
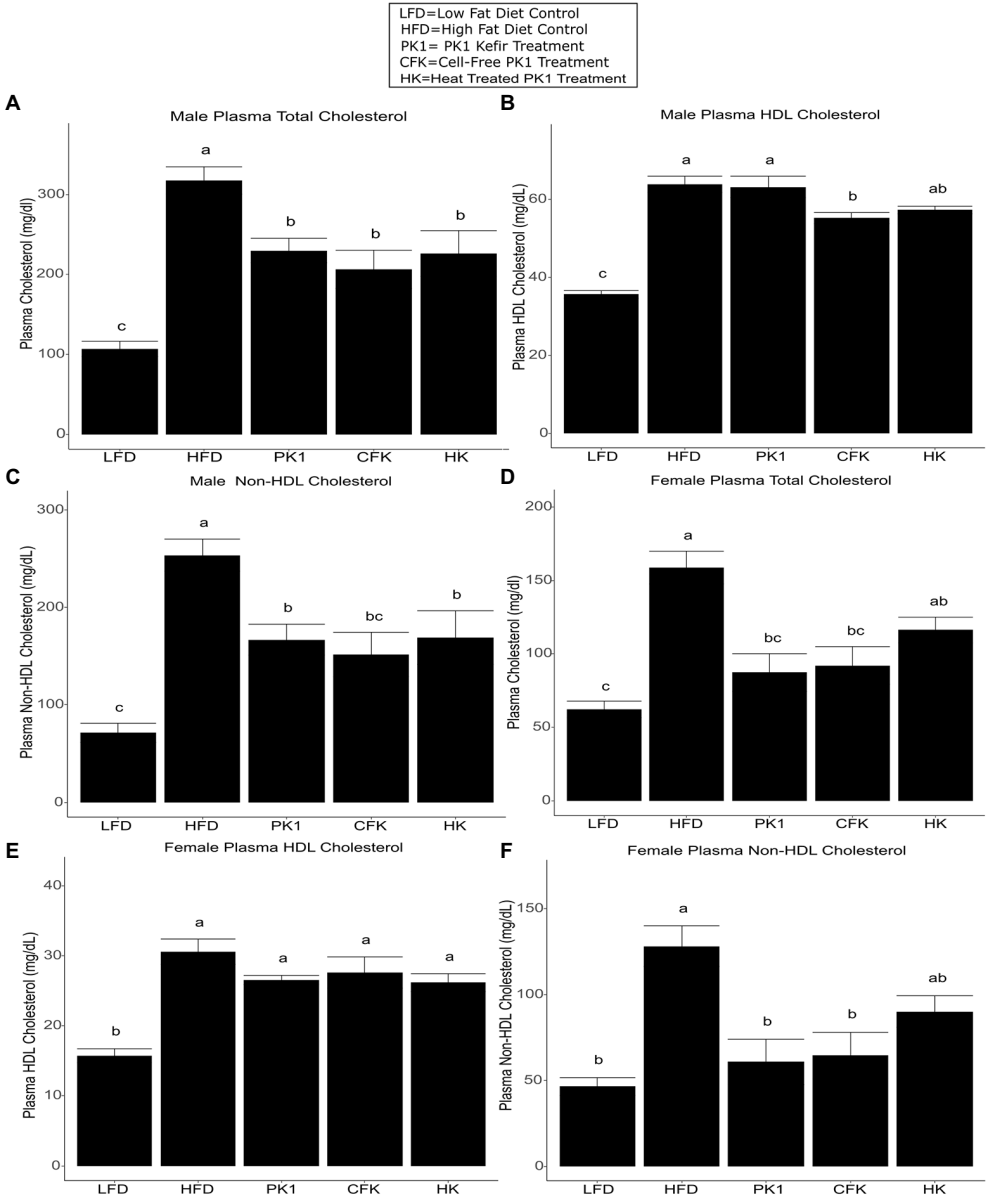
Concentration of total plasma cholesterol **(A,D)**, HDL cholesterol **(B,E)**, and non-HDL cholesterol **(C,F)** in male/female C57Bl/6 mice fed a control diet or high-fat diet supplemented with milk or different kefir preparations for 8 weeks. Data are expressed as mean values with their standard errors (*n* = 7–8). Means that do not share a letter are significantly different (*p* < 0.05). LFD, mice fed a low fat diet supplemented with freeze-dried milk for 8 weeks; HFD, mice fed a high fat diet supplemented with freeze-dried milk for 8 weeks; PK1, mice fed a high fat diet supplemented with freeze-dried pitched kefir for 8 weeks; CFK, mice fed a high-fat diet supplemented with freeze-dried cell-free kefir; HK, mice fed a high-fat diet supplemented freeze-dried heat-treated kefir.

Total cholesterol levels were examined in order to determine how different kefir fractions impact cholesterol metabolism. In male mice, each of the PK1, CFK, and HK groups had lower total cholesterol levels than the HFD control group (Figure 2A), while in female mice the PK1 and CFK groups had lower levels of total cholesterol and HK mice showed a trend for a reduction (*p* = 0.054; Figure 2D). HDL cholesterol was unchanged among all high-fat diet fed groups of female mice (Figure 2E). In male mice, while there was no difference between HFD, PK1, and HK groups, the HDL cholesterol levels in the CFK group were lower than both HFD and PK1 (Figure 2B). Non-HDL cholesterol followed the same pattern as total cholesterol in both sexes with all treatment groups being lower in the male mice (Figure 2C), while PK1 and CFK were lower in females; however, HK mice no longer showed a trend to be lower than HFD (Figure 2F).

### Only whole kefir lowered liver lipid levels

**Figure 3:**
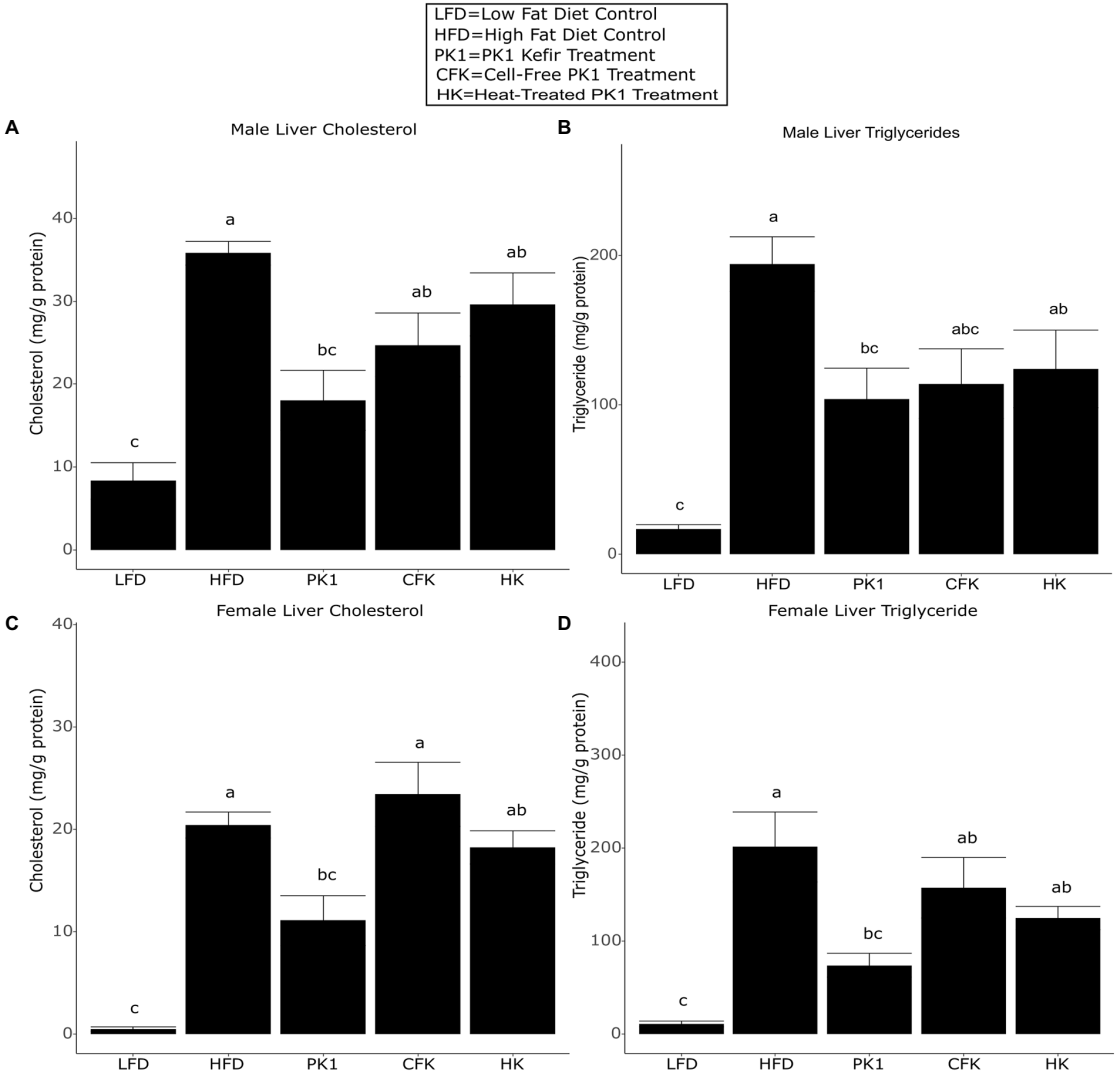
Concentration of liver cholesterol **(A,C)** and triglycerides **(B,D)** in male/female C57Bl/6 mice fed a control diet or high-fat diet supplemented with milk or different kefir preparations for 8 weeks. Data are expressed as mean values with their standard errors (*n* = 7–8). Means that do not share a letter are significantly different (*p* < 0.05). LFD, mice fed a low fat diet supplemented with freeze-dried milk for 8 weeks; HFD, mice fed a high fat diet supplemented with freeze-dried milk for 8 weeks; PK1, mice fed a high fat diet supplemented with freeze-dried pitched kefir for 8 weeks; CFK, mice fed a high-fat diet supplemented with freeze-dried cell-free kefir; HK, mice fed a high-fat diet supplemented freeze-dried heat-treated kefir.

Liver triglyceride and cholesterol levels are common indicators of metabolic health and NAFLD and as such, we sought to determine how consumption of different kefir fractions impacted these measures. In male mice, PK1 consumption lowered both liver cholesterol (Figure 3A) and triglyceride (Figure 3B) content when compared to HFD control, while CFK showed a trend to lower triglyceride levels (*p* = 0.090; Figure 3B) and HK was not different for either measure. In female mice, PK1 once again resulted in a decrease in liver cholesterol (Figure 3C) and triglycerides (Figure 3D), while HK and CFK consumption did not alter levels of liver triglycerides or cholesterol.

### Kefir treatments have varied effects on circulating markers of CVD risk

**Figure 4:**
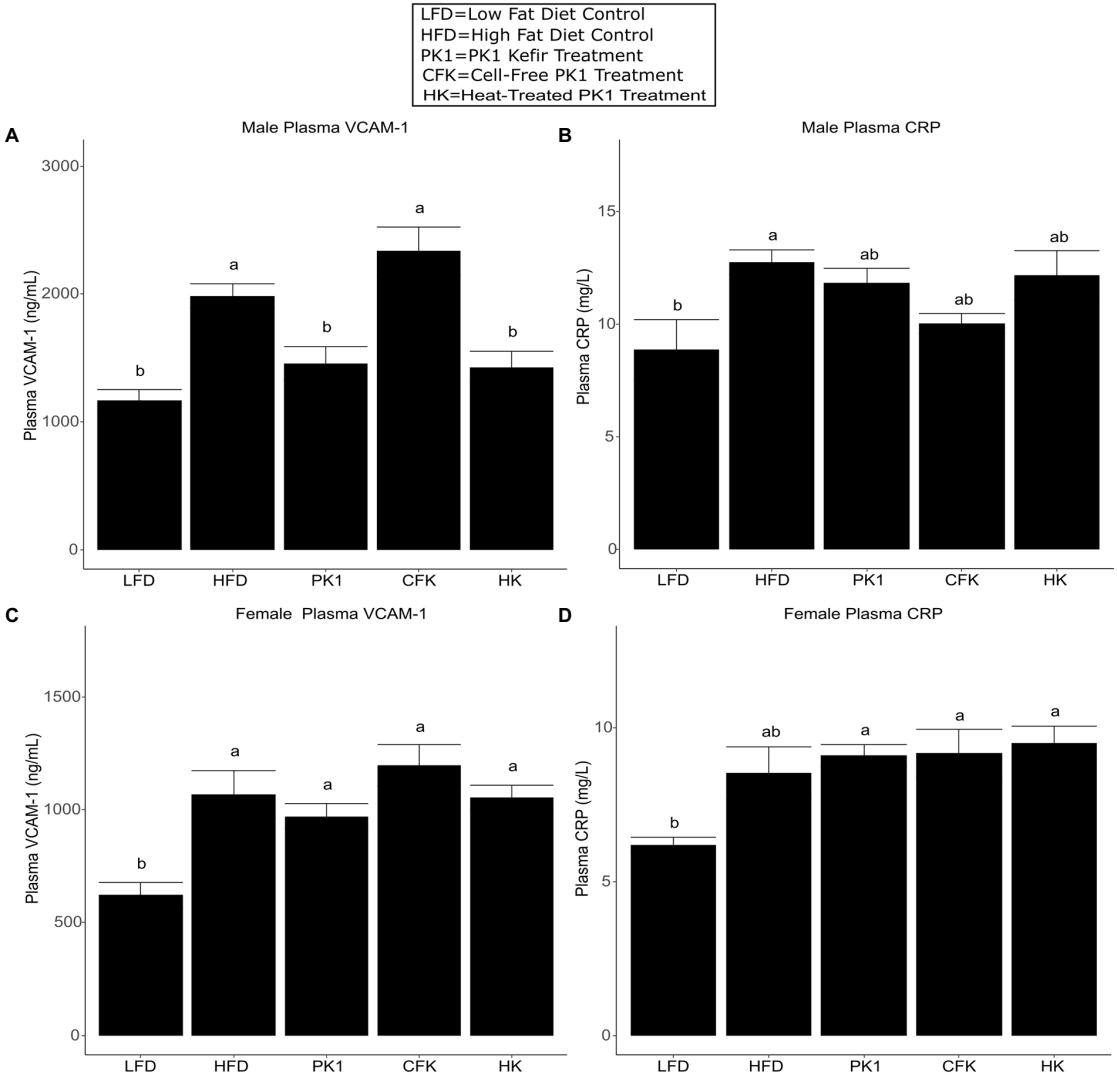
Concentration of plasma VCAM-1 **(A,B)** and CRP **(C,D)** in male/female C57Bl/6 mice fed a control diet or high-fat diet supplemented with milk or different kefir preparations for 8 weeks. Data are expressed as mean values with their standard errors (*n* = 7–8). Means that do not share a letter are significantly different (*p* < 0.05). LFD, mice fed a low fat diet supplemented with freeze-dried milk for 8 weeks; HFD, mice fed a high fat diet supplemented with freeze-dried milk for 8 weeks; PK1, mice fed a high fat diet supplemented with freeze-dried pitched kefir for 8 weeks; CFK, mice fed a high-fat diet supplemented with freeze-dried cell-free kefir; HK, mice fed a high-fat diet supplemented freeze-dried heat-treated kefir.

As high plasma cholesterol levels are related to the development of CVD, we determined how different kefir preparations impacted circulating levels of VCAM-1 and CRP, which are important factors in the development of atherosclerosis and common biomarkers of CVD risk. In male mice, both PK1 and HK resulted in a reduction in plasma VCAM-1 (Figure 4A); however, this was not seen in female mice (Figure 4B). In male mice, plasma CRP was elevated in response to HFD (*p* < 0.05) relative to LFD, whereas all kefir treatments were intermediate and not different from LFD or HFD (Figure 4C). In female mice, all kefir treatments had higher plasma CRP than LFD, and were not different from HFD (Figure 4D).

### Kefir supplementation did not alter short-chain fatty acid composition in mice cecum

Cecal content analysis of SCFAs found no difference in kefir supplemented treatment groups compared to HFD control (Table 1). The LFD group maintained the greatest amount of acetate, propionate, and butyrate concentrations compared to HFD control in both female and male mice. Total SCFA concentrations were significantly higher for both female (*p* < 0.001) and male (*p* < 0.01) mice of the LFD group compared to the HFD group.

**Table 1:** Concentration of SCFAs (μmol/g) in cecal content of male and female C57Bl/6 mice fed a control diet or high-fat diet supplemented with milk or different kefir preparations for 8 weeks.

SCFA (μmol/g)	LFD	HFD	CFK	HK	PK1
*Female*					
Acetate	23.64 ± 1.56***	11.54 ± 1.68	13.17 ± 5.92	13.46 ± 3.58	12.14 ± 3.35
Propionate	12.39 ± 1.30***	7.35 ± 0.90	7.46 ± 2.42	8.04 ± 2.33	6.97 ± 1.58
Butyrate	6.08 ± 1.32***	1.67 ± 0.56	1.98 ± 1.73	2.13 ± 0.85	1.45 ± 0.84
Total SCFAs	42.92 ± 2.96***	20.9 ± 2.06	23.05 ± 10.16	24.04 ± 6.03	20.87 ± 5.14
*Male*					
Acetate	16.84 ± 5.31#	11.73 ± 1.23	12.63 ± 1.93	12.74 ± 2.71	13.87 ± 2.6
Propionate	10.29 ± 3.03	9.67 ± 0.89	9.08 ± 1.66	9.27 ± 1.35	11.23 ± 1.99
Butyrate	6.77 ± 1.34***	1.24 ± 0.26	1.54 ± 0.29	1.12 ± 0.25	1.33 ± 0.15
Total SCFAs	34.6 ± 8.76**	23.23 ± 1.89	23.71 ± 3.39	23.61 ± 3.73	27.07 ± 4.35

Data are expressed as mean values with their standard errors (*n* = 7–8). **Indicates a significantly different value from HFD (*p* < 0.01), ***indicates *p* < 0.001. LFD, mice fed a low-fat diet supplemented with freeze-dried milk for 8 weeks; HFD, mice fed a high fat diet supplemented with freeze-dried milk for 8 weeks; PK1, mice fed a high fat diet supplemented with freeze-dried pitched kefir for 8 weeks; CFK, mice fed a high-fat diet supplemented with freeze-dried cell-free kefir; HK, mice fed a high-fat diet supplemented freeze-dried heat-treated kefir.

### Kefir had no impact on overall microbial community structure

**Figure 5:**
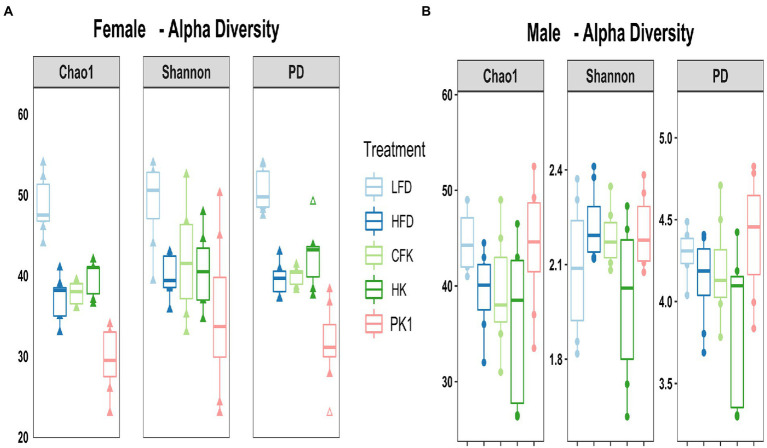
Alpha diversity metrics of the cecal microbiota in male/female C57Bl/6 mice fed a control diet or high-fat diet supplemented with milk or different kefir preparations for 8 weeks. **(A)** Chao1 and PD indices indicated higher microbial diversity and richness in the LFD group (*p* < 0.05) and lower diversity in the Kefir group (*p* < 0.05) compared to HFD, CFK and HK groups for females with no difference with Shannon diversity index other than LFD maintained higher diversity. **(B)** No difference was found in male mice after kefir consumption for Chao1, Shannon or PD metrics compared to HFD group (*n* = 7–8). Means that do not share a letter are significantly different (*p* < 0.05). LFD, mice fed a low fat diet supplemented with freeze-dried milk for 8 weeks; HFD, mice fed a high fat diet supplemented with freeze-dried milk for 8 weeks; PK1, mice fed a high fat diet supplemented with freeze-dried pitched kefir for 8 weeks; CFK, mice fed a high-fat diet supplemented with freeze-dried cell-free kefir; HK, mice fed a high-fat diet supplemented freeze-dried heat-treated kefir.

**Figure 6:**
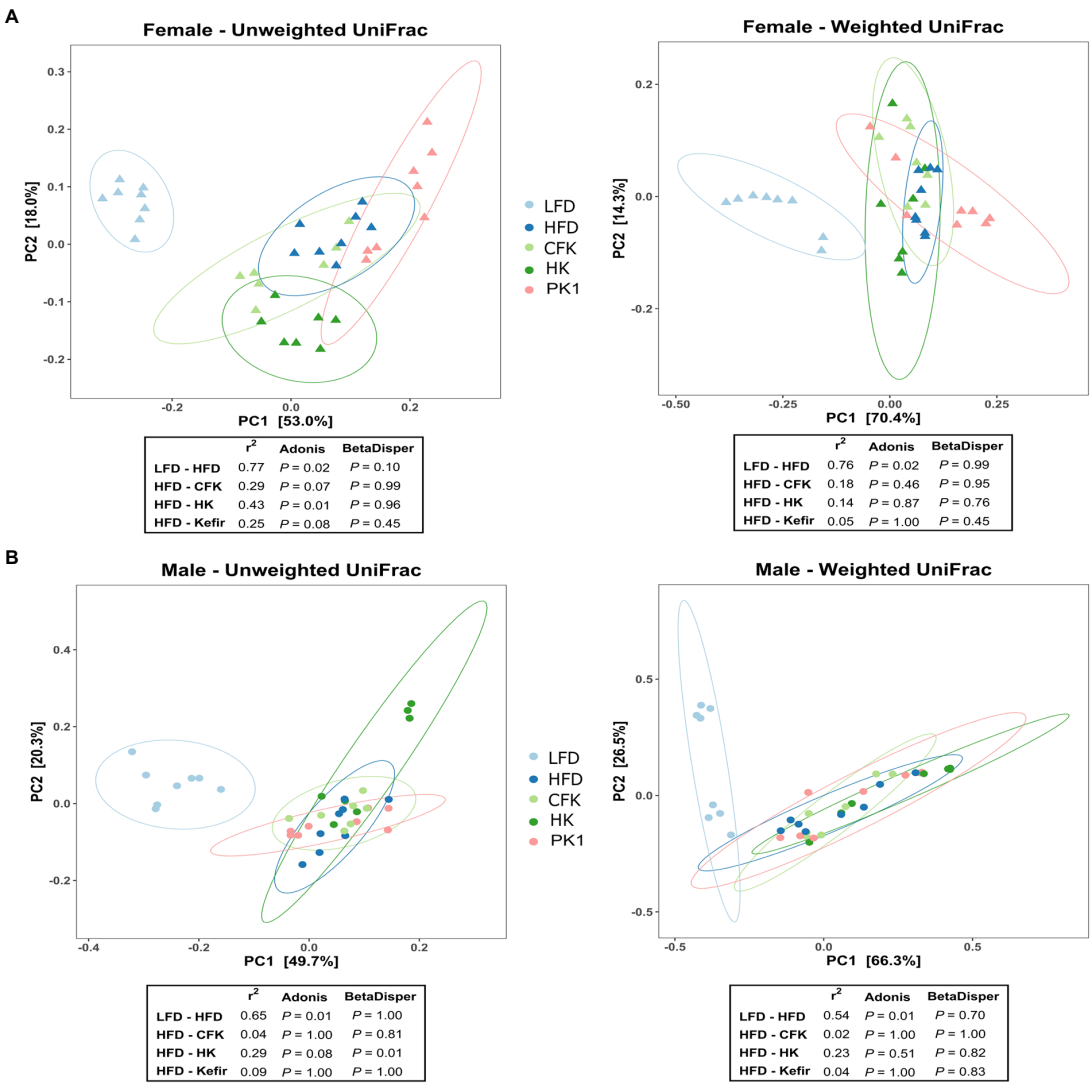
Principle coordinate analysis using unweighted and weighted UniFrac distance metric of the cecal microbiota of male/female C57Bl/6 mice fed a control diet or high-fat diet supplemented with milk or different kefir preparations for 8 weeks. A major shift between LFD and HFD groups was determined in both **(A)** female and **(B)** male mice with no difference between HFD and kefir consumption groups (CFK, HK and Kefir; *n* = 7–8). Means that do not share a letter are significantly different (*p* < 0.05). LFD, mice fed a low fat diet supplemented with freeze-dried milk for 8 weeks; HFD, mice fed a high fat diet supplemented with freeze-dried milk for 8 weeks; PK1, mice fed a high fat diet supplemented with freeze-dried pitched kefir for 8 weeks; CFK, mice fed a high-fat diet supplemented with freeze-dried cell-free kefir; HK, mice fed a high-fat diet supplemented freeze-dried heat-treated kefir.

Cecal microbial analysis revealed that supplementation with kefir preparations on a HFD (Kefir, HK, and CFK) had no appreciable impact on microbial community structure (Figures 5, 6). For female mice, alpha diversity metrics Chao1 and PD were significantly higher for the LFD group (*p* < 0.05) and lower for the PK1 group (*p* < 0.05) relative to the HFD, HK, and CFK groups. Shannon diversity was greatest for LFD, which was lower for HFD and PK1 groups (Figure 5A). The Chao1, Shannon and PD alpha diversity indices for male mice were not considerably impacted (Figure 5B). Weighted and unweighted UniFrac distance metrics all showed that the high fat diet accounted for the major shifts in microbial communities found in the cecum following kefir consumption when compared to the LFD group in both female and male mice (unweighted *p* < 0.02 and *p* < 0.01; weighted *p* < 0.02 and *p* < 0.01 respectively).

## Discussion

This is the first study to examine how different kefir preparations consisting of whole kefir, cell-free kefir, and heat-treated kefir impact plasma and liver lipid profiles, the gastrointestinal microbiome, and plasma markers of CVD risk in both males and females using a mouse model of obesity. Given that previous work on traditional kefir had shown that multiple different fractions had positive health impacts (Bourrie et al., 2016), we expected that the CFK and HK treatments in our study would improve lipid and CVD biomarker profiles similarly to PK1 in both male and female mice. Male mice showed a reduction in weight gain across all three kefir treatment groups, while female mice did not have a reduction across any of the treatment groups relative to HFD. Interestingly, both plasma total cholesterol levels were lowered by all kefir preparations in both males and females, while HDL cholesterol was not different among all kefir treatments in females and was only lower in CFK male mice when compared to the HFD control. Non-HDL cholesterol levels were also lower in each kefir treatment in male mice, and in all but the HK group in female mice. This is important as elevated total and non-HDL cholesterol levels are associated with an increased risk of CVD and other metabolic diseases (Després and Lemieux, 2006; Brunner et al., 2019). We also observed that only PK1 and HK reduced VCAM-1 in males. However, this was not seen in the female mice. This may be due to the increased weight gain and generally more severe phenotypes observed in male C57Bl/6 mice when compared to their female counterparts receiving HFD. Indeed, male mice gained approximately 40% more weight than females when fed HFD and VCAM-1 levels in male HFD mice were nearly twice as high as in their female counterparts, while the male LFD group had VCAM-1 levels that were nearly identical to the HFD group in female mice.

In addition to metabolic syndrome and CVD, NAFLD is associated with hyperlipidemia and can lead to steatosis and liver cancer (Loomba and Sanyal, 2013; Duan et al., 2014; Younossi et al., 2018), with increased levels of liver lipids associated with NAFLD risk (Angulo, 2002). We found that liver cholesterol and triglyceride levels were improved by PK1 in both male and female mice. The CFK treatment exhibited a trend for reduction of liver triglycerides in male mice only, and HK did not reduce liver cholesterol or triglycerides in either sex. These results are interesting, as they are contradictory to what we observed in plasma lipid profiles and may indicate that the component of kefir responsible for the observed reduction in liver lipids is somehow removed or reduced during the CFK and HK kefir preparation. One potential reason for these findings may relate to the angiotensin-converting enzyme (ACE) inhibitory action of kefir, which has been attributed to peptides produced from casein during fermentation (Quiros et al., 2005). ACE inhibitory drugs have been found to improve circulating cholesterol and lipid profiles in both human and animal studies (Ravid et al., 1995; Vasiljević et al., 1999; Dost et al., 2014). Some evidence suggests that these drugs may prevent fibrosis and cancer in existing NAFLD; however, more studies are needed, and no studies have examined if ACE inhibitors can prevent the development of NAFLD (Jonsson et al., 2001; Moreno et al., 2010; Li et al., 2018). It is therefore possible that the component of kefir responsible for lowering plasma cholesterol levels is some sort of heat stable peptide with ACE inhibitory activity, while there is a separate component responsible for the improvements in liver lipid levels that is lost or reduced during the preparation of CFK and HK.

Elevated plasma cholesterol, particularly elevated non-HDL cholesterol, has been associated with an increased risk of developing both CVD and diabetes (Scherer and Hill, 2016; Francula-Zaninovic and Nola, 2018; Brunner et al., 2019). One common underlying factor in CVD is the development of atherosclerosis and the associated plaque formation, which can increase an individual’s risk of an adverse cardiovascular event (Francula-Zaninovic and Nola, 2018). Cell adhesion molecules, such as VCAM-1, are commonly used as biomarkers of endothelial function and have been used as indicators of increased CVD and atherosclerosis risk (Springer, 1994; Meng et al., 2021) and play an important role in plaque development (Cybulsky et al., 2001; Ley and Huo, 2001; Varona et al., 2019; Troncoso et al., 2021). We observed a reduction in plasma VCAM-1 in male mice fed both PK1 and HK, but not in the CFK while female mice showed no differences in VCAM-1 between any of the HFD fed groups. The sex difference is likely a result of the less severe atherosclerotic phenotype in female C57BL/6 mice (Paigen et al., 1987), with females in our trial having roughly half the level of VCAM-1 as males. A previous study found that VCAM-1 and plaque formation in the aorta were reduced by a peptide enriched spray dried kefir in HFD fed *ApoE^−/−^* mice (Tung et al., 2020). It is possible that the phenotypes observed in this study would have been more pronounced had we used a more severe phenotype, such as the *ApoE^−/−^* mouse. The lack of effect from CFK treatment may indicate that the compound responsible for this benefit is lost during the centrifugation and filtration process used to create cell-free kefir. Systemic inflammation is another important factor contributing to CVD risk (Vandanmagsar et al., 2011; Boulangé et al., 2016), with CRP being regarded as an important indicator of CVD event risk (Backes et al., 2004; Bisoendial et al., 2010; Avan et al., 2018). Our study found that CRP was not reduced by any of the kefir preparations in either male or female mice fed HFD. This may indicate that, while kefir is able to impact endothelial function positively, the active components associated with these benefits do not impact chronic systemic inflammation as it relates to CRP levels. It is also important to note that, in mice, CRP is only a modest acute phase protein and may not respond to inflammation to the same degree as in humans (Torzewski et al., 2014; Huang et al., 2019). This may be why there has been evidence in human trials that kefir consumption can improve plasma CRP levels despite no corresponding evidence from mouse trials (Ghizi et al., 2021).

All three kefir preparations supplemented to a high-fat and high-cholesterol diet minimally affected the cecal microbiota compared to the high-fat diet alone. PK1 consumption did not increase alpha diversity, as has been observed in a recent study exploring fermented food consumption in a human trial (Wastyk et al., 2021). Absence of an effect on alpha diversity in the current study could be explained by the background diet (HFD vs. uncontrolled) and the controlled environment used for mouse experiments. Due to the lack of fermentable carbohydrates, a high-fat diet can significantly alter gut microbial structure and decrease SCFA concentrations compared to a standard low-fat diet (Dalby et al., 2017). A change in SCFA levels is well documented to correlate with gastrointestinal health and microbiota (Makki et al., 2018; Cong et al., 2022). In agreement, we found higher SCFAs concentrations in the cecum of LFD group with normal cardiovascular health profiles along with changes to the microbial community compared to HFD group. These differences may be due to fermentable fiber content of the low fat and high fat diets, as the low fat diet is made from whole food ingredients containing a variety of fibers while the high fat diet is semi-purified with cellulose as the only fiber. The fact that all three kefir fractions were unable to alter SCFA concentrations or the microbiota suggests that the beneficial effects on cholesterol levels in mice fed kefir is likely not related to SCFA production in the gut. The slight difference in the low abundance microbes in the cecum of female mice but not male mice, as determined by unweighted UniFrac analysis, is likely a result of sex differences in weight, food consumption, locomotor activity, energy expenditure and β cell adaptation upon dietary manipulation (Casimiro et al., 2021). It is also important to note that, while our results did not show significant changes to the cecal microbiome associated with kefir consumption, the composition of the gastrointestinal microbiome of C57BL/6 mice has been shown to differ throughout the gastrointestinal tract (Lkhagva et al., 2021). It is therefore possible that kefir consumption impacts microbiome composition in other sections of the gastrointestinal tract that may contribute to the physiological impacts observed in this study.

This study expands on previous work showing that a pitched kefir (PK1) containing traditional kefir microbes can improve plasma and liver lipid profiles in a mouse model of obesity and that different components of kefir can have beneficial impacts on host health (Maeda et al., 2005; Uchida et al., 2010; Bourrie et al., 2021), however this is the first study to examine different fractions of a pitched kefir in an attempt to differentiate the active components related to lipid control and atherosclerosis improvements. This is also the first study to our knowledge to examine these types of health benefits in both male and female mice, which is of particular importance given the sex differences that exist in both mice and humans with respect to the metabolic responses to obesity and the development of associated metabolic diseases (Paigen et al., 1987; Matyšková et al., 2008; Kautzky-Willer and Handisurya, 2009; Hwang et al., 2010; Sugiyama and Agellon, 2012; Yang et al., 2014). We found that while weight gain was lowered by PK1 consumption in male mice only, each of the kefir treatments lowered plasma total cholesterol and non-HDL cholesterol when compared to a HFD control in both sexes; however, only PK1 treatment resulted in a reduction in liver triglycerides and cholesterol levels in both male and females. These results are interesting as they show that consumption of either cell-free or heat-treated kefir results in similar improvements to plasma cholesterol profiles when compared to PK1 consumption but that improvements in liver lipids requires whole PK1 kefir. This may indicate that live kefir microorganisms are necessary to lower liver lipid content. Additionally, the impact of the kefir treatments for plasma and liver lipids was consistent across males and females, indicating that the mechanism of action is not sex dependent. VCAM-1 was only lowered in male mice receiving PK1 or heat-treated kefir, indicating a possible sex dependent response for this particular biomarker. It is also important to note that female mice had nearly half the levels of VCAM-1 when compared to male mice, and it is possible that with a more severe obesity phenotype there would have been a comparable decrease in plasma VCAM-1 between males and females. Additionally, the lack of improvement in VCAM-1 levels in CFK mice may indicate that, while live microbes are not necessary to lower VCAM-1 in plasma, their microbial components are. These results provide confirmation that reconstituted PK1 kefir containing specific kefir microbes can improve lipid profiles as well as circulating markers of atherosclerosis, and that the lipid lowering benefits are present in both males and females. This is encouraging, as the potential for functional foods that equally benefit male and female individuals is especially important given the increase in females developing metabolic diseases and the need for more research examining these conditions in female populations (Engin, 2017). Future work should focus on identifying how these fractions differ in the composition of their fermentation metabolites and, if possible, on the identification of an active component or components that are responsible for the health benefits observed in these animal trials.

## Data availability statement

The datasets presented in this study can be found in online repositories. The names of the repository/repositories and accession number(s) can be found below: BioProject, PRJNA885841.

## Ethics statement

The animal study was reviewed and approved by Animal Care and Use Committee at the University of Alberta (AUP 00000671).

## Author contributions

BCTB, PDC, CR, and BPW designed research. BCTB, AJF, and TJ conducted research. BCTB and AJF analyzed data. BCTB, AJF, and BPW wrote the paper. BPW had primary responsibility for final content. All authors have read and approved the final manuscript.

## Funding

This study was directly supported by a grant from the Weston Family Microbiome Initiative. BPW and CR are supported by the Canada Research Chairs Program. Outside of this work, CR also reports grants from the Canadian Institute of Health Research (CIHR), the Natural Sciences and Engineering Research Council of Canada (NSERC) and several other agencies. Cotter laboratory is funded by Science Foundation Ireland (SFI) under grant number SFI/12/RC/2273 (APC Microbiome Ireland), by SFI together with the Irish Department of Agriculture, Food and the Marine under grant number SFI/16/RC/3835 (VistaMilk), and by the European Commission under the Horizon 2020 program grant number 818368 (MASTER). Research in the Willing lab is supported by a Natural Sciences and Engineering Discovery Grant (RGPIN-2019-06336).

## Conflict of interest

BCTB, BPW, and PDC hold a patent for the method used to produce the pitched kefir used in the trial.

The remaining authors declare that the research was conducted in the absence of any commercial or financial relationships that could be construed as a potential conflict of interest.

## Publisher’s note

All claims expressed in this article are solely those of the authors and do not necessarily represent those of their affiliated organizations, or those of the publisher, the editors and the reviewers. Any product that may be evaluated in this article, or claim that may be made by its manufacturer, is not guaranteed or endorsed by the publisher.
